# Combined analysis of inorganic elements and flavonoid metabolites reveals the relationship between flower quality and maturity of *Sophora japonica* L.

**DOI:** 10.3389/fpls.2023.1255637

**Published:** 2023-11-17

**Authors:** Tian-Wang Wang, Jun Tan, Long-Yun Li, Yong Yang, Xiao-Mei Zhang, Ji-Rui Wang

**Affiliations:** ^1^ Three Grade Laboratory of Chinese Medicine Chemistry, Chongqing Academy of Chinese Materia Medica, Chongqing, China; ^2^ Chongqing Sub-Center of National Resource Center for Chinese Materia Medica, China Academy of Chinese Medical Sciences, Chongqing, China

**Keywords:** flavonoid metabolites, inorganic elements, maturation, quality change, *Sophora japonica*

## Abstract

Flos Sophorae (FS), or the dried flower buds of Sophora japonica L., is widely used as a food and medicinal material in China. The quality of S. japonica flowers varies with the developmental stages (S1–S5) of the plant. However, the relationship between FS quality and maturity remains unclear. Inductively coupled plasma optical emission spectrometry (ICP-OES) and ultra-high performance liquid chromatography coupled with electrospray ionization-triple quadrupole-linear ion trap mass spectrometry (UPLC-ESI-Q TRAP-MS/MS) were used to analyze inorganic elements and flavonoid metabolites, respectively. A combined analysis of the inorganic elements and flavonoid metabolites in FS was conducted to determine the patterns of FS quality formation. Sixteen inorganic elements and 173 flavonoid metabolites that accumulated at different developmental stages were identified. Notably, 54 flavonoid metabolites associated with the amelioration of major human diseases were identified, and Ca, P, K, Fe, and Cu were postulated to influence flavonoid metabolism and synthesis. This study offers a novel perspective and foundation for the further exploration of the rules governing the quality of plant materials.

## 1 Introduction

Flos Sophorae (FS; Huaihua in Chinese), or the dried flower buds of *Sophora japonica* L. (Leguminosae), is widely distributed in East and Southeast Asia. It is extensively used in the industrial extraction of rutin, which possesses important physiological and pharmacological properties, such as anti-inflammatory (Liu et al., 2022), anti-ultraviolet B radiation (Li et al., 2019), and antitumor properties (Zhong et al., 2022). FS is edible, so it is used as a food pigment and additive to improve the appearance and taste of food (Chen et al., 2010). Moreover, it is often used to treat various diseases, including hemorrhoids, dizziness, and hypertension; its medicinal value is attributed to its flavonoid content (He et al., 2016). Flavonoids are important for improving immune function against human diseases and for regulating plant growth by improving pollen activity, enhancing plant adaptability to unfavorable environmental conditions, and protecting plants against herbivores (Ma et al., 2018; Dai et al., 2019; An et al., 2020; Grunewald et al., 2020; Daryanavard et al., 2023). Flavonoids are polyphenolic compounds with a benzo-γ-pyrone structure and are divided into seven main subclasses: flavones, flavonols, isoflavones, flavanols, flavanones, anthocyanins, and chalcones (Shen et al., 2022). The biological activities of flavonoids are structure dependent; for example, aglycones are more effective antioxidants than their corresponding glycosides (Yuan et al., 2022), and flavonoids with free 3-OH groups are excellent antioxidants (Tao et al., 2023). Extensive research has been conducted on rutin, quercetin, isorhamnetin, and kaempferol in FS (Liu et al., 2016; Li et al., 2022; Li et al., 2023; Zhang et al., 2023); however, few studies have been conducted on the other flavonoid components of FS due to their low content and more intricate structures. The variations in the flavonoid composition of *S. japonica* during its growth (Wang et al., 2019) leads to changes in the pharmaceutical efficacy of FS.

The inorganic elements and flavonoid metabolites in FS are key indicators of their quality, which serve important nutritional functions in the human body (Lötscher et al., 2022; Genchi et al., 2023) and affect the growth and metabolism of the plant itself. Inorganic elements, which increase flavonoid synthesis and the ability of plants to withstand stress, stimulate the activities of phenylalanine ammonia-lyase (PAL), chalcone isomerase (CHI), and chalcone synthase (CHS) (Jiao et al., 2016). Previous research on FS has mainly focused on its pharmacological and chemical composition (He et al., 2016; Li et al., 2019; Liu et al., 2022). Hence, limited information is available on the relationship between inorganic elements and FS quality; the changes in the inorganic elements of FS during the different growth stages remains unclear. Therefore, the comparative identification of the inorganic elements and flavonoid metabolites of FS during flower maturity could provide a new perspective and basis for exploring the quality formation rules of *S. japonica*.

Inductively coupled plasma–optical emission spectrometry (ICP–OES), which is characterized by low detection limits and high precision to simultaneously determine multiple elements, has been widely used to analyze inorganic elements in food and herbal medicines (Mohamed et al., 2022; Oyebayo et al., 2022). As a rapid and precise analytical method for the qualitative and quantitative analyses of small-molecule metabolites using high-throughput chemical analysis techniques, metabolomics has been widely applied in nutritional sciences (Tebani and Bekri, 2019), toxicology (Yen et al., 2023), and plant metabolism research (Zhao et al., 2022). With the advancement of analytical techniques, metabolomics has become an essential tool for investigating plant physiology, particularly plant development (Wang C. et al., 2022) and stress resistance mechanisms (Qin et al., 2022).

In this study, the inorganic elements and flavonoid metabolites in FS were determined to analyze the quality variations in the flower maturity of *S. japonica*. The results of this study can provide a valuable basis for the proper harvesting of FS.

## 2 Materials and methods

### 2.1 Chemicals and reagents

Inorganic element standards were purchased from AccuStandard (New Haven, CT, USA). Nitric acid (HNO_3_), potassium iodide (KI), and ascorbic acid (ASA) were purchased from the Chuandong Chemical Group (Chongqing, China), Kelong Chemical (Chengdu, China), and Xilong Chemical (Guangdong, China), respectively. High-performance-liquid-chromatography-grade methanol, acetonitrile, and ethanol were procured from Merck (Darmstadt, Germany).

### 2.2 Plant material

**Figure 1:**
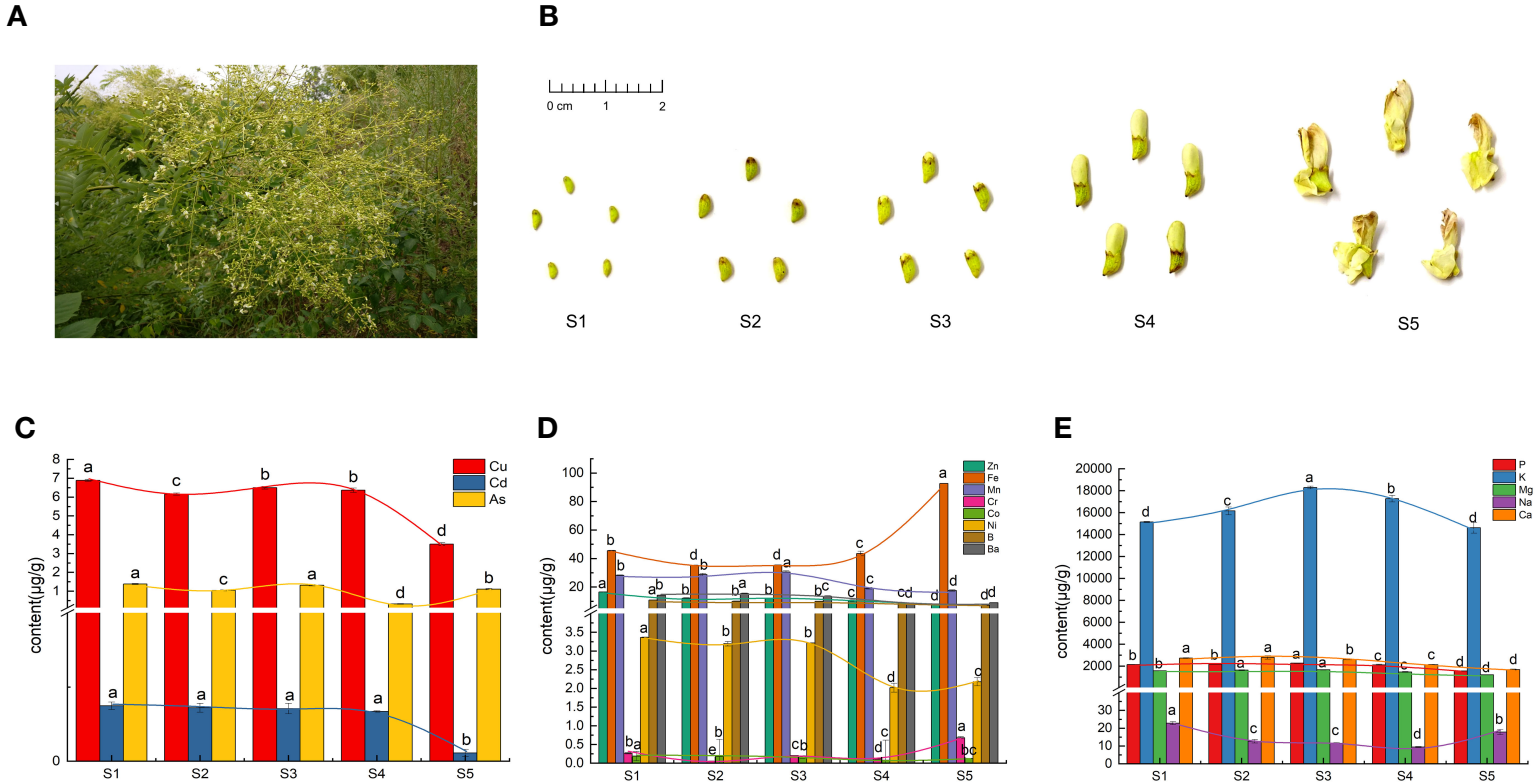
Phenotypic features and inorganic elements in Flos Sophorae (FS). **(A)** Inflorescence morphology of *S. japonica*. **(B)** Phenotypic features of FS in the five developmental stages (S1–S5). **(C)** Heavy-metal elements. **(D)** Trace elements. **(E)** Macroelements. Different letters indicate significant differences (*p* < 0.05).

The flowers and flower buds used in this study were harvested from an *S. japonica* plantation (**Figure 1A**) in the Dazu district (29°56′N, 105°68′E), Chongqing, China, at an altitude of 379 m. The specimens were identified by Li Longyun, a researcher from the Chongqing Academy of Chinese Materia Medica. The specimens were categorized into five developmental stages (S1–S5) based on the differences in their characteristics. The classification criteria used in this study were consistent with those used in a previous study (Wang et al., 2019). The phenotypic features of the FS specimens are shown in **Figure 1B**. All the samples were divided into two aliquots, frozen in liquid nitrogen, and stored at −80°C until their further use for inorganic elements determination and metabolomics analysis.

### 2.3 Determination of inorganic elements

The inorganic elements in the FS specimens were determined according to the method described by Cui et al. (2018). Six milliliters of concentrated HNO_3_ was added to 0.2 g of the FS samples and pre-digested at 100°C for 1 h. Afterwards, the pre-digestion solution was prepared in a microwave digester (CEM, NC, USA), following the optimized program (**Table S1**). The digest was evaporated to 2 mL on a thermostatic heater at 100°C. After cooling, the digest was diluted to 20 mg/mL with ultrapure water to detect the macroelements (P, K, Mg, Na, and Ca), trace elements (Zn, Fe, Mn, Cr, Co, Ni, B, Mo, V, Se, and Ba), and selected heavy-metal elements (Cu, Pb, and Cd). Subsequently, 0.5 mL of 10% KI (freshly prepared just before use) and 0.5 mL of 5% ASA (freshly prepared just before use) were mixed with 3 mL of the sample diluent and heated at 100°C for 20 min to detect As and Hg. An ICP-OES instrument (Perkin Elmer, CT, USA) was employed for detecting all the inorganic elements in the FS samples. The detection procedures are presented in **Table S2**.

### 2.4 Metabolomic analysis

The methods for metabolite extraction, detection, and analysis were based on a previous study (Wang J. R. et al., 2022). The FS samples were freeze-dried and crushed with zirconia beads at 30 Hz for 1.5 min using a mixer mill (MM 400; Retsch, Haan, Germany). Specifically, 100 mg of the lyophilized FS was weighed and extracted with 1 mL of 70% methanol at 4°C overnight. After extraction, the supernatant was obtained by centrifugation at 10,000 × *g* for 10 min. The supernatants were further cleaned through solid-phase extraction (CNWBOND Carbon-GCB SPE Cartridge, 250 mg, 3 mL; ANPEL, Shanghai, China) before being passed and used for ultra-high performance liquid chromatography coupled with electrospray ionization-triple quadrupole-linear ion trap mass spectrometry (UPLC-ESI-Q TRAP-MS/MS) analysis. Quality control samples were prepared by combining aliquots from all the individual samples.

The extracts were analyzed using a UPLC-ESI-Q TRAP-MS/MS system equipped with a C18 column (Waters Acquity UPLC HSS T3 C18, 2.1 × 100 mm, 1.8 µm). Ultrapure water (0.04% acetic acid) was used as mobile phase A, and acetonitrile (0.04% acetic acid) was used as mobile phase B. The gradient program was as follows: A/B 95:5 (v/v) at 0 min, 5:95 (v/v) at 11.0 min, 5:95 (v/v) at 12.0 min, 95:5 (v/v) at 12.1 min, and 95:5 (v/v) at 15.0 min; flow rate of 0.4 mL/min; column temperature at 40°C; and injection volume of 5 μL. The electrospray ionization (ESI) source operation parameters were as follows: turbo spray as the ion source, source temperature at 550°C, ion spray voltage at 5,500 V, and curtain gas at 25.0 psi.

### 2.5 Identification of the active pharmaceutical ingredients of FS related to the resistance to eight major diseases

To identify the bioactive ingredients in the FS samples, the names of the flavonoid metabolites derived from the UPLC-ESI-Q TRAP-MS/MS analysis were entered into the chemical name menu of the Traditional Chinese Medicine Systems Pharmacology Database and Analysis Platform (https://old.tcmsp-e.com/tcmsp.php). We conducted screening to identify the bioactive ingredients associated with the prevention and treatment of 8 major diseases in humans, including cancer/tumors, diabetes, hypertension, cardiovascular diseases, atherosclerosis, thrombotic diseases, hepatitis, and hemorrhoids.

### 2.6 Data analysis

All metabolites were annotated using the MetWare database and quantified using multiple reaction monitoring. The metabolite data were analyzed using Analyst 1.6.1. The data were log2-transformed for statistical analysis to enhance the normality of the distribution. The distribution and accumulation characteristics of flavonoid metabolites at the five developmental stages were visualized by performing principal component analysis (PCA), hierarchical clustering analysis (HCA), and K-means cluster analysis. PCA was performed to minimize the dimensionality of the multidimensional data, visualize the trend of metabolome separation among the groups, and assess the presence of differences among the sample groups. K-means cluster analysis was performed to identify trends in flavonoid metabolites. The differential flavonoid metabolites were screened through orthogonal partial least squares–discriminant analysis (OPLS–DA) with two screening criteria: variable importance in projection value ≥ 1 and fold change value ≥ 2 or FC ≤ 0.5. OPLS–DA is a multivariate statistical analysis method with supervised pattern recognition that can maximize group differentiation and help to find differentially expressed metabolites. Volcano and Venn plots were prepared using the R software to visualize the results. Statistical significance was determined by performing one-way analysis of variance followed by Duncan’s test in SPSS version 20.0.

The screened flavonoid metabolites were annotated by referring to the Kyoto Encyclopedia of Genes and Genomes (KEGG) database (https://www.kegg.jp/). Subsequently, the annotated metabolites were drawn on a pathway map. The role of inorganic elements in synthesizing flavonoid metabolites was investigated by reviewing the relevant literature. The results were imported into Cytoscape 3.7.2 to create a visual network diagram of “inorganic elements—differential metabolites–metabolic pathways”.

## 3 Results

### 3.1 Inorganic elements in FS

As shown in **Figures 1C–E**, the Fe, Cr, Ni, and Na concentrations of the FS samples initially decreased then increased, with the lowest accumulation at S2 and S4. Conversely, the Ba, Mg, Mn, P, and K concentrations of the FS samples initially increased then decreased, with the highest accumulation at S2 and S3. The accumulation of Cd, Zn, Co, B, and Ca gradually decreased as FS further developed. The accumulation of Cu was significantly higher than that of the other heavy elements at all stages, whereas the accumulation of As exhibited an undulating pattern, reaching the highest level at S1 and the lowest level at S4. Mercury and lead were not detected in any of the five stages.

### 3.2 Metabolite detection in FS

**Figure 2:**
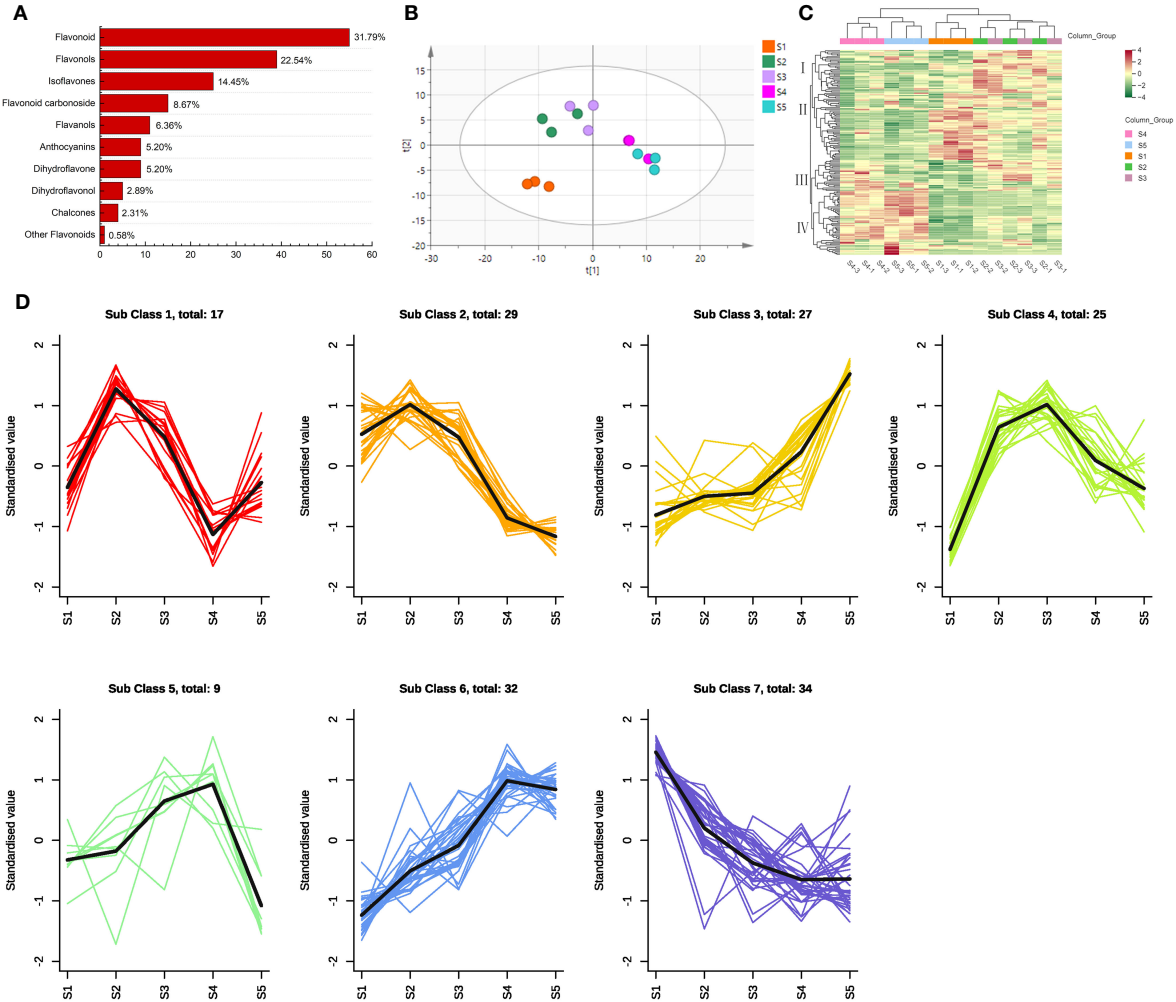
Multivariate statistical analysis of the flavonoid metabolites in different flower maturity of *S. japonica*. **(A)** Classification of flavonoid metabolite structure. **(B)** Principal component analysis of the flavonoid metabolites. **(C)** Hierarchical cluster analysis of the flavonoid metabolites. **(D)** K-means cluster analysis of the flavonoid metabolites.

A total of 173 flavonoid metabolites were identified from the FS samples (**Figure 2A**; **Table S3**): flavonoids (55), flavonols (39), isoflavones (25), flavonoid carbonosides (15), flavanols (11), anthocyanins (9), dihydroflavones (9), dihydroflavonols (5), chalcones (4), and other flavonoids (1).

Based on the PCA results, we identified two principal components (PC1 and PC2) that represented 42.8% and 17.7% of the total variance, respectively (**Figure 2B**). The samples were then divided into three groups. S1 was categorized as a separate group, indicating that the flavonoid metabolites changed dramatically during the early stages of bud maturation. S2 and S3 were grouped together, and similar results were obtained from the analysis of all secondary metabolites (Wang J. R. et al., 2022), indicating a substantial contribution of flavonoid metabolites to the changes in all secondary metabolites during this period. S4 and S5 were closely distributed, indicating that the differences in flavonoid metabolites between S4 and S5 were not significant.

According to the HCA results (**Figure 2C**), the replicate samples of S2 and S3 were grouped along the abscissa axis, which is consistent with the PCA results. Notably, S4 and S5 were clustered into two classes although they were highly similar in terms of the expression intensity of flavonoid metabolites. The four groups were categorized along the ordinate axis based on the accumulation of flavonoid metabolites. Category I metabolites exhibited the highest accumulation levels at S2 and S3 and the lowest levels at S4 and S5. Meanwhile, Category II metabolites exhibited the highest accumulation levels at S1 and the lowest levels at S4 and S5. Furthermore, Category III metabolites accumulated at the highest levels in S2, S3, and S4, whereas Category IV flavonoid metabolites accumulated at the highest levels in S4 and S5.

Based on the results of the K-means cluster analysis, the 173 flavonoid metabolites identified were classified into seven subclasses (**Figure 2D**; **Table S4**). The majority of the dihydroflavones (7/9) and chalcones (2/4) in Subclasses 2 and 7 exhibited a declining trend from S1 to S5, whereas those in Subclasses 3 and 6 exhibited an increasing trend throughout this period. The concentration of flavonoid metabolites in Subclasses 4 and 5 initially increased then decreased; the highest concentrations were recorded at S3 and S4, respectively. The accumulation of 17 flavonoid metabolites in Subclass 1 peaked at S2 but rapidly declined at S4.

### 3.3 Identification of the active pharmaceutical ingredients in FS for resistance to eight major diseases

The eight major diseases affecting humans worldwide are cancers/tumors, diabetes, hypertension, cardiovascular diseases, atherosclerosis, thrombotic diseases, hepatitis, and hemorrhoids. Although flavonoid metabolites are the key therapeutic components of FS, their contribution to the treatment of these 8 major diseases remains unclear. A total of 54 flavonoid metabolites responsible for resistance to at least one of the 8 major diseases were identified (**Table 1**). In total, 54, 35, 46, 52, 27, 24, 15, and 45 metabolites exhibited anticancer, antidiabetic, antihypertensive, anti-cardiovascular, anti-atherosclerotic, anti-thrombotic, anti-hepatitis, and anti-hemorrhoid properties, respectively. Several metabolites were associated with resistance to multiple diseases. For instance, quercetin, genistein, daidzein, biochanin A, formononetin, jaceosidin, isoliquiritigenin, isorhamnetin, and trifolin were associated with resistance to all eight major diseases. Kaempferol and rutin were associated with resistance to seven diseases, and narcissin was associated with resistance to two diseases; these three metabolites are the primary flavonoid components of FS.

**Table 1:** List of 54 disease-resistance flavonoid metabolites identified in Flos Sophorae.

Class	Anti-cancer/tumor ingredients	Anti-diabetic ingredients	Anti-hypertensive ingredients	Anti-cardiovascular ingredients	Anti-atherosclerotic ingredients	Anti-thrombotic ingredients	Anti-hepatitis ingredients	Anti-hemorrhoid ingredients
**Flavonols (15)**	Quercitrin	Quercitrin	Quercitrin	Quercitrin	–	Quercitrin	–	–
	Hyperin	Hyperin	Hyperin	Hyperin	–	–	–	Hyperin
	Isoquercitrin	Isoquercitrin	–	Isoquercitrin	–	Isoquercitrin	–	–
	Protocatechuic acid	Protocatechuic acid	Protocatechuic acid	Protocatechuic acid	–	–	–	Protocatechuic acid
	Kaempferol	Kaempferol	Kaempferol	Kaempferol	Kaempferol	Kaempferol	–	Kaempferol
	Gossypitrin	Gossypitrin	–	–	–	–	–	–
	Avicularin	–	Avicularin	Avicularin	–	Avicularin	–	Avicularin
	Quercetin	Quercetin	Quercetin	Quercetin	Quercetin	Quercetin	Quercetin	Quercetin
	Juglanin	–	Juglanin	Juglanin	–	Juglanin	–	Juglanin
	Narcissin	–	–	Narcissin	–	–	–	–
	Rutin	Rutin	Rutin	Rutin	Rutin	–	Rutin	Rutin
	Isorhamnetin	Isorhamnetin	Isorhamnetin	Isorhamnetin	Isorhamnetin	Isorhamnetin	Isorhamnetin	Isorhamnetin
	Trifolin	Trifolin	Trifolin	Trifolin	Trifolin	Trifolin	Trifolin	Trifolin
	Astragalin	Astragalin	Astragalin	Astragalin	Astragalin	Astragalin	–	Astragalin
	Sexangularetin	Sexangularetin	Sexangularetin	Sexangularetin	–	–	–	Sexangularetin
**Isoflavones (12)**	Orobol	–	–	–	–	–	–	–
	Genistein	Genistein	Genistein	Genistein	Genistein	Genistein	Genistein	Genistein
	Prunetin	Prunetin	Prunetin	Prunetin	Prunetin	Prunetin	–	Prunetin
	Daidzein	Daidzein	Daidzein	Daidzein	Daidzein	Daidzein	Daidzein	Daidzein
	Biochanin A	Biochanin A	Biochanin A	Biochanin A	Biochanin A	Biochanin A	Biochanin A	Biochanin A
	3’-Methoxydaidzein	3’-Methoxydaidzein	3’-Methoxydaidzein	3’-Methoxydaidzein	3’-Methoxydaidzein	3’-Methoxydaidzein	–	3’-Methoxydaidzein
	Formononetin	Formononetin	Formononetin	Formononetin	Formononetin	Formononetin	Formononetin	Formononetin
	Glycitein	Glycitein	Glycitein	Glycitein	Glycitein	Glycitein	–	Glycitein
	Glycitin	–	Glycitin	Glycitin	Glycitin	Glycitin	Glycitin	Glycitin
	Calycosin	Calycosin	Calycosin	Calycosin	Calycosin	Calycosin	–	Calycosin
	Ononin	Ononin	Ononin	Ononin	Ononin	Ononin	–	Ononin
	Isoformononetin	Isoformononetin	Isoformononetin	Isoformononetin	Isoformononetin	Isoformononetin	–	Isoformononetin
**Flavonoids (7)**	Diosmetin	Diosmetin	Diosmetin	Diosmetin	–	–	–	Diosmetin
	Acacetin	Acacetin	Acacetin	Acacetin	–	–	Acacetin	Acacetin
	Luteolin	Luteolin	Luteolin	Luteolin	–	–	Luteolin	Luteolin
	Sophoricoside	–	Sophoricoside	Sophoricoside	–	–	–	–
	Diosmin	Diosmin	Diosmin	Diosmin	–	–	–	Diosmin
	Luteolin-7-O-glucoside	–	Luteolin-7-O-glucoside	Luteolin-7-O-glucoside	–	–	–	Luteolin-7-O-glucoside
	Apigenin	Apigenin	Apigenin	Apigenin	–	Apigenin	–	Apigenin
**Flavanols (7)**	Epigallocatechin gallate	Epigallocatechin gallate	Epigallocatechin gallate	Epigallocatechin gallate	Epigallocatechin gallate	–	Epigallocatechin gallate	Epigallocatechin gallate
	Gallic acid	Gallic acid	Gallic acid	Gallic acid	–	–	Gallic acid	Gallic acid
	(-)-Epicatechin gallate	–	–	(-)-Epicatechin gallate	(-)-Epicatechin gallate	–	–	–
	Catechin	–	Catechin	Catechin	Catechin	–	–	Catechin
	(-)-Epigallocatechin	–	(-)-Epigallocatechin	(-)-Epigallocatechin	(-)-Epigallocatechin	–	–	(-)-Epigallocatechin
	(-)-Epiafzelechin	–	(-)-Epiafzelechin	(-)-Epiafzelechin	(-)-Epiafzelechin	–	–	(-)-Epiafzelechin
	Methyl gallate	–	Methyl gallate	Methyl gallate	–	–	–	Methyl gallate
**Dihydroflavone (5)**	Eriodictyol	–	–	Eriodictyol	–	–	–	Eriodictyol
	Liquiritigenin	–	Liquiritigenin	Liquiritigenin	Liquiritigenin	–	–	Liquiritigenin
	Narirutin	–	–	Narirutin	–	–	–	–
	Naringenin-7-O-glucoside	–	Naringenin-7-O-glucoside	Naringenin-7-O-glucoside	–	–	–	Naringenin-7-O-glucoside
	Butin	–	Butin	Butin	–	–	–	Butin
**Anthocyanins (4)**	Jaceosidin	Jaceosidin	Jaceosidin	Jaceosidin	Jaceosidin	Jaceosidin	Jaceosidin	Jaceosidin
	Cyanidin chloride	–	Cyanidin chloride	Cyanidin chloride	–	–	–	Cyanidin chloride
	Peonidin	–	Peonidin	Peonidin	–	Peonidin	–	Peonidin
	Pelargonidin	Pelargonidin	Pelargonidin	Pelargonidin	Pelargonidin	–	–	Pelargonidin
**Flavonoid carbonoside (2)**	Puerarin	Puerarin	Puerarin	Puerarin	Puerarin	–	–	Puerarin
	Genistein 8-C-glucoside	Genistein 8-C-glucoside	–	Genistein 8-C-glucoside	–	–	–	–
**Dihydroflavonol (1)**	Taxifolin	Taxifolin	Taxifolin	Taxifolin	–	–	–	Taxifolin
**Chalcones (1)**	Isoliquiritigenin	Isoliquiritigenin	Isoliquiritigenin	Isoliquiritigenin	Isoliquiritigenin	Isoliquiritigenin	Isoliquiritigenin	Isoliquiritigenin

### 3.4 Differential flavonoid metabolites in the five flower maturity of *S. japonica*

**Figure 3:**
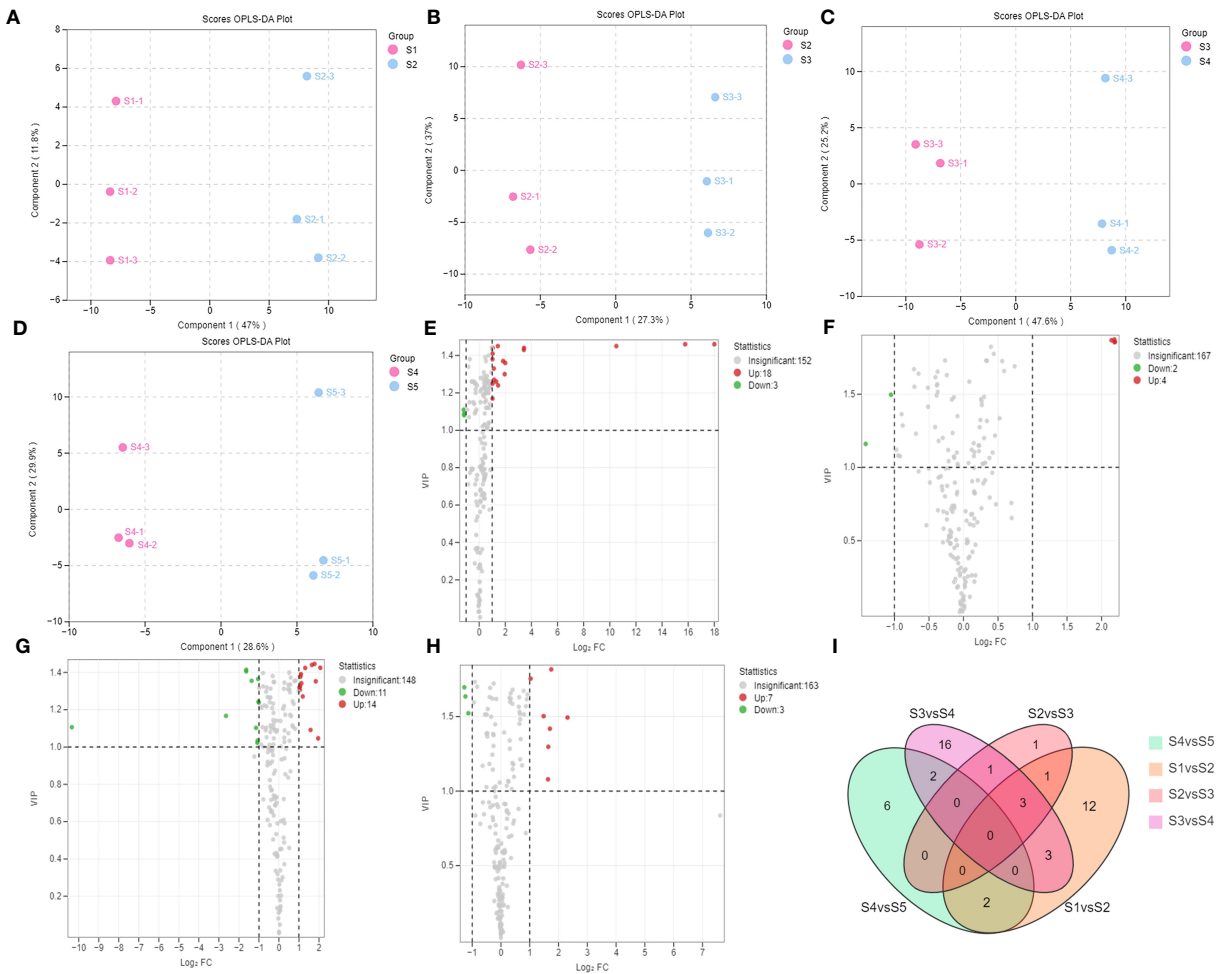
Differential flavonoid metabolite analysis of the samples in the five developmental stages of *S. japonica*. **(A–D)** Orthogonal partial least squares-discriminant analysis model plots for the following comparisons: S1 vs. S2, S2 vs. S3, S3 vs. S4, and S4 vs. S5, respectively. **(E–H)** Volcano plots show the expression levels of the differential flavonoid metabolites in the comparisons S1 vs. S2, S2 vs. S3, S3 vs. S4, and S4 vs. S5, respectively. Each dot represents a metabolite: red dots indicate upregulated flavonoid metabolites; green dots indicate downregulated flavonoid metabolites; and black dots indicate flavonoid metabolites detected with insignificant expression differences. **(I)** The Venn diagram indicates the common and unique metabolites in the comparison groups.

Differences between the groups of flavonoid metabolites were compared using the OPLS-DA model (**Figures 3A–D**, **S1A–F**), where Q2 represents the prediction ability. The Q2 scores in all pairwise comparisons were higher than 0.836 (S1 vs. S2, 0.933; S2 vs. S3, 0.836; S3 vs. S4, 0.914; S4 vs. S5, 0.89; S1 vs. S3, 0.948; S1 vs. S4, 0.985; S1 vs. S5, 0.981; S2 vs. S4, 0.96; S2 vs. S5, 0.967; and S3 vs. S5, 0.938), indicating that the models were appropriate. Differences in the expression levels of the metabolites were visualized using volcano plots. The differential flavonoid metabolites screened between the adjacent stages are shown in **Figures 3E–H**; **Table S5**. The differential flavonoid metabolites screened between the non-adjacent stages are shown in **Figures S1G–L**; **Table S6**. The unique differential flavonoid metabolites present in each adjacent stage comparison group are shown in **Figure 3I**.

### 3.5 Enrichment analysis and metabolic pathway analysis

When comparing adjacent stages (**Figures S2A–D**), there was notable enrichment in pathways related to isoflavonoid biosynthesis (ko00943), anthocyanin biosynthesis (ko00942), flavonoid biosynthesis (ko00941), and flavone and flavonol biosynthesis (ko00944). Between the non-adjacent stages (**Figures S2E–J**), isoflavonoid biosynthesis (ko00943) was highly enriched in S1 vs. S3 and S1 vs. S4; flavone and flavonol biosynthesis (ko00944) was highly enriched in S1 vs. S5, S2 vs. S5, and S3 vs. S5; and flavonoid biosynthesis (ko00941) was highly enriched in S2 vs. S4.

**Figure 4:**
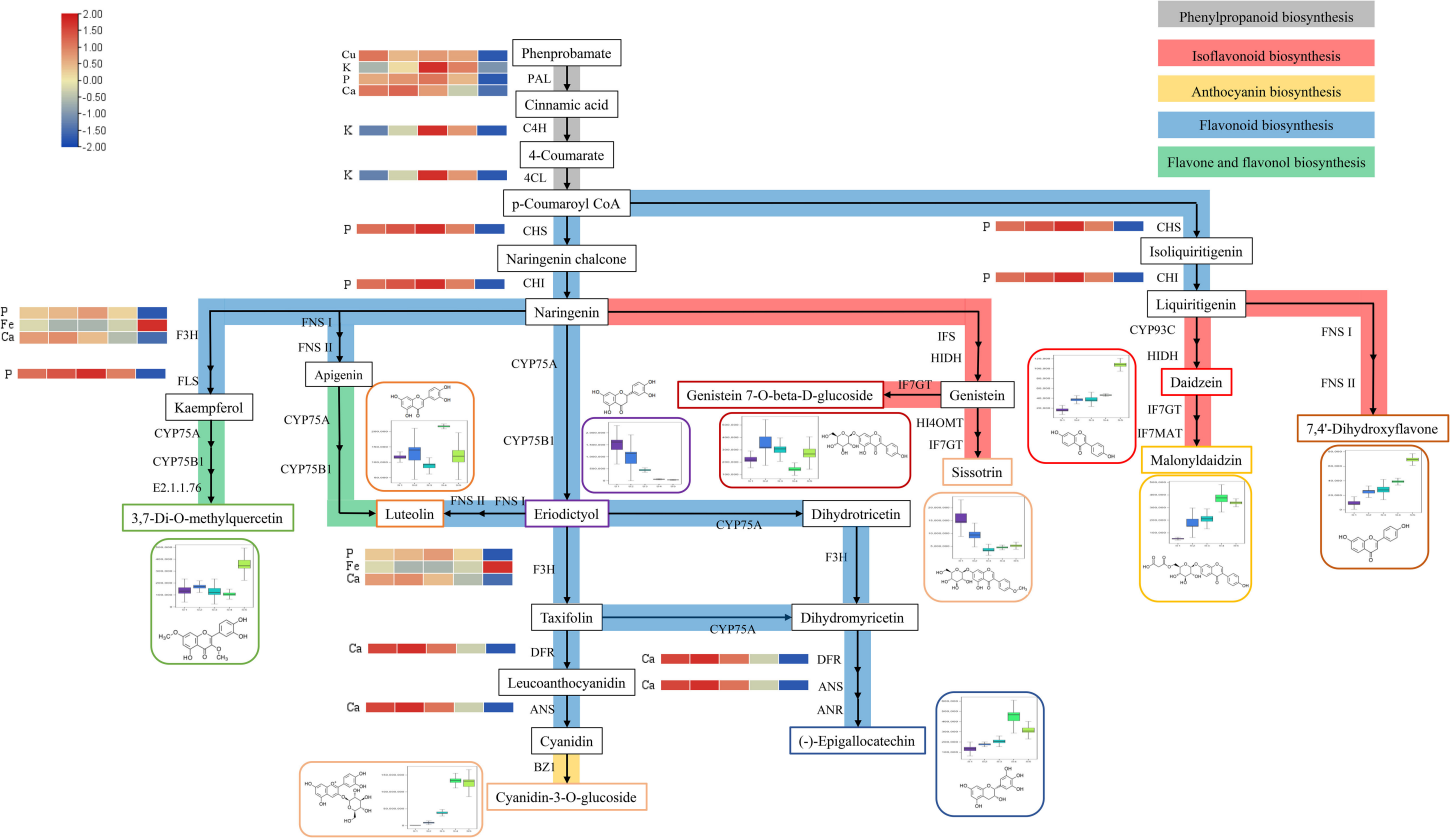
Partial pathway map of the differential flavonoid metabolites in Flos Sophorae. Grey represents the phenylpropanoid synthesis pathway; red represents the isoflavone synthesis pathway; yellow represents the anthocyanin synthesis pathway; blue represents the flavonoid synthesis pathway; and green represents the flavone and flavonol synthesis pathway.

The partial synthetic pathways of the flavonoid metabolites were mapped according to the results of KEGG enrichment analysis. As shown in **Figure 4**, 7,4’-dihydroxyflavone, daidzein, genistein 7-O-beta-D-glucoside, sissotrin, and malonyldaidzin are involved in isoflavonoid biosynthesis. Cyanidin-3-O-glucoside is present in anthocyanin biosynthesis. Luteolin, eriodictyol, and (-)-epigallocatechins are involved in flavonoid biosynthesis. 3,7-Di-O-methylquercetin and luteolin are involved in flavone and flavonol biosynthesis. Notably, naringin and eriodictyol are located at the intersection of multiple synthetic pathways, indicating that they play an important role in the synthesis of flavonoid metabolites.

**Figure 5:**
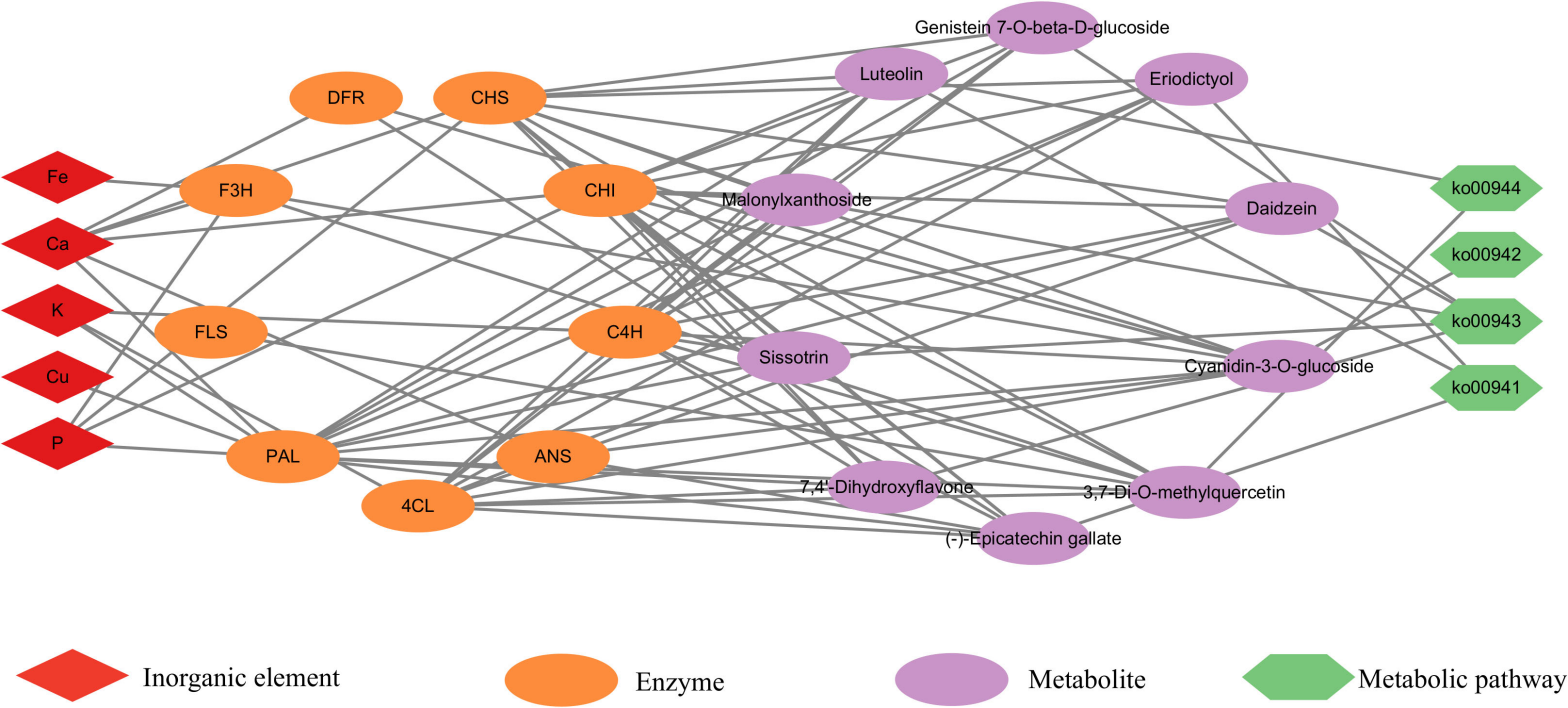
Inorganic elements–differential metabolites–metabolic pathways correlation network. The red diamond represents an inorganic element; the orange ellipse represents an enzyme; the purple ellipse represents differential metabolite; and the green hexagon represents a metabolic pathway.

According to the literature, Ca, P, K, Fe, and Cu play important roles in the synthesis of flavonoid metabolites synthesis pathway (Li et al., 2014; Jia et al., 2015; Jiao et al., 2016; GonzÁLez-Mendoza et al., 2018; Sun et al., 2019). Ten differential flavonoid metabolites and five elements were imported into Cytoscape 3.7.2 software to construct a visual network diagram of the inorganic elements, differential metabolites, and metabolic pathways (**Figure 5**). The regulatory effects of P, Fe, and Ca on flavonoid 3-hydroxylase and flavonol synthase can affect the concentrations of 3,7-Di-O-methylquercetin and cyanidin-3-O-glucoside. Furthermore, Ca had a regulatory effect on dihydroflavonol 4-reductase and anthocyanin synthase, which influenced the cyanidin-3-O-glucoside and (-)-epigallocatechin content. Notably, Cu, K, P, and Ca regulate PAL, cinnamate 4-hydroxylase (C4H), and 4-coumaric acid-CoA ligase (4CL), which are active in the phenylpropanoid biosynthesis pathway. As an upstream synthesis pathway, the influence of phenylpropanoid biosynthesis may be transmitted to other pathways (isoflavonoid biosynthesis, anthocyanin biosynthesis, flavonoid biosynthesis, and flavone and flavonol biosynthesis), affecting all 10 flavonoid metabolites.

## 4 Discussion

### 4.1 Changes in the inorganic elements of FS in different stages

Inorganic elements are closely associated with human health. Macroelements have been reported to improve immunity (Lötscher et al., 2022), regulate blood pressure (Hornig et al., 2023), and promote bone growth (Nair et al., 2023). Trace elements promote enzyme production and ensure the normal functioning of the endocrine system (Shi et al., 2020). Various inorganic elements were identified in the FS, including P, K, Mg, Na, and Ca. Most macroelements and trace elements were highly enriched during the small-bud stages (S1, S2, and S3) and decreased during the blooming stage. Consequently, early-harvested flowers may have more potent medicinal benefits (Wang et al., 2019). However, flowers harvested at the later stages may also be useful medicinal materials because of the accumulation of other ingredients. For example, the high Fe concentration at S5 can promote hemoglobin synthesis and induce therapeutic effects against anemia (Charles-Edwards et al., 2019). The concentration of heavy metals is one of the factors affecting the quality of an edible and medicinal material. Heavy metals As, Cu, and Cd, which can be harmful to human health when consumed in excessive amounts (Bryan et al., 2019; Mahmood et al., 2023), were highly enriched at S1. At S1, the buds were not fully developed and had a low yield (**Table S7**). Therefore, flowers at S2 to S3 are more suitable for harvesting as edible materials (Tian et al., 2022).

In addition, inorganic elements are important for plant growth and development. Cu and Ca have been shown to promote PAL, CHI, and CHS activities, consequently enhancing isoflavone synthesis (Jiao et al., 2016; GonzÁLez-Mendoza et al., 2018). K activates various enzymes by promoting ATP generation (Li et al., 2014). In this study, it was found that an increased isoflavone concentration was accompanied by an increased quantity of inorganic elements (K, P, and Ca) from S1 to S2. Therefore, we speculated that K, P, and Ca play a role in the metabolic pathway of isoflavones during the early growth stages, resulting in elevated levels of isoflavonoid metabolites (genistein 7-O-beta-D-glucoside and daidzein). The enrichment of isoflavones can improve the adaptability of plants to unfavorable environments, including low temperatures, UV radiation, and heavy-metal stress (Ma et al., 2018; Veremeichik et al., 2021; Gu et al., 2022). This suggests that the application of K and P fertilizers during the development of *S. japonica* can enhance its environmental adaptability (Liu et al., 2017).

### 4.2 Differences in the flavonoid metabolites of FS in different stages

Flavonoid metabolites are crucial for the quality of FS. In this study, 173 flavonoid metabolites were identified at the five growth stages. In the early stages of flower development (S1–S2), the type and content of flavonoids were significantly enriched, indicating that these components are closely related to the physiological activities of the flowers (Chakraborty et al., 2022). Anthocyanin was the main flavonoid metabolite that changed from S2 to S3, consistent with previous reports (Guo et al., 2021). As the anthocyanin content increased, the color of the calyx changed from lime green to yellowish green. Flavonols, flavonoids, and anthocyanins were enriched in the middle stages of flower maturity. The accumulation of these components benefits plant growth and development by enhancing pollen vitality and reducing insect damage by inhibiting insect development (Liu et al., 2023; Xue et al., 2023). During the opening period of the corolla, the anthocyanin content of *S. japonica* is further enriched, contributing to the bright color of the flowers (Guo et al., 2021). The upregulation of flavones and isoflavones during this period can enhance the adaptability of plants to stressors (Deng et al., 2019; Li et al., 2020). In contrast, most dihydroflavones exhibited a declining trend during flowering, which could be attributed to the fact that the flavonoid synthesis pathway is located upstream of the anthocyanin synthesis pathway. Zhang et al. (2017) also found that enzymes catalyzed by flavonoids in *S. japonica* had a competitive effect on the synthesis of anthocyanins.

### 4.3 Identification of the active pharmaceutical ingredients in FS for the resistance to 8 major diseases

Rutin is the major medicinal ingredient in FS and is used as an indicator to evaluate the quality of FS in the Chinese Pharmacopoeia (Chinese Pharmacopoeia Commission, C.P., 2020). Its beneficial effects on treating hypertension, hepatitis, cardiovascular disease, and atherosclerosis are consistent with the observation that FS cools blood for hemostasis and clears liver fires. Additionally, quercetin, genistein, daidzein, biochanin A, formononetin, jaceosidin, isoliquiritigenin, isorhamnetin, and trifolin exhibited therapeutic properties against 8 major diseases, indicating that these metabolites are the most important active pharmaceutical ingredients of FS for promoting human health. Consequently, FS can be considered an ideal food for preventing these diseases (Song et al., 2023).

As shown in **Table 1**, the most abundant active ingredients of FS with therapeutic effects were flavonol and isoflavonoid metabolites. Tao et al. (2023) reported that the addition of a glycoside at the C-3 position of quercetin-3-O-galactoside increased the bond dissociation enthalpy of the phenolic hydroxyl groups, resulting in weakened antioxidant activity. Furthermore, Yuan et al. (2022) found that the antioxidant activity of aglycones is stronger than that of their glucosides and vitamin C. When the 3-OH group of flavonols is replaced by glycoside groups, steric hindrance impedes the binding of the inhibitor to the active site of the enzyme (Li et al., 2023). This phenomenon was verified in another study, in which the FS extract showed better inhibition of tyrosinase activity after treatment with a weak acid than before treatment (Lai et al., 2014). Generally, the results indicate that the 3-OH group of flavonols plays an important role in antioxidants (Xiao et al., 2021). Isoflavones also exhibit excellent antioxidant properties. The diverse substitution patterns on the C ring and complex structural relationships among different moieties contribute to the remarkable antioxidant activities of isoflavones (Castellano and Torrens, 2015), which are beneficial for treating various diseases (Neha et al., 2019).

### 4.4 Synthetic pathways of the partial flavonoid metabolites

Metabolite synthesis is a complex process influenced by multiple interacting factors. As shown in **Figure 4**, naringenin and eriodictyol are at the intersection of multiple pathways, and the accumulation of metabolites is influenced by multiple pathways (Saxena et al., 2023; Zheng et al., 2023). Metabolites usually accumulate in the opposite direction if they are in a competitive interaction with a common precursor. For example, 3,7-di-O-methylquercetin and luteolin demonstrated opposite accumulation trends from S4 to S5 because they share a precursor component (naringenin) and compete for flavone and flavonol biosynthesis. Flavone synthase Is and flavanone 3β-hydroxylases can catalyze the conversion of naringin to apigenin and dihydrokaempferol, which are upstream components of 3,7-di-O-methylquercetin and luteolin, respectively (Han et al., 2014; Fu et al., 2023). The same phenomenon was observed for genistein 7-O-beta-D-glucoside and sissotrin in the isoflavonoid biosynthesis from S1 to S2. Due to the activation of hydroxyindole-o-metal transfer, the 4'-OH on the B ring of genistein is replaced by methoxy groups, resulting in different downstream metabolites (Kudapa et al., 2023). Although cyanidin-3-O-glucoside and (-)-epigallocatechin share a common precursor (taxifolin), they exhibited a consistent accumulation tendency, indicating that they are involved in other metabolic pathways (**Figure 4**: dihydrotricetin → dihydromyricetin) that lead to changes in their concentrations. Interestingly, the downstream products of liquiritigenin, daidzein, and 7,4'-dihydroxyflavone also exhibit a consistent cumulative trend. The difference lies in whether the B ring is located at the 2^nd^ or 3^rd^ position of the chromogenic ketone, which depends on the activation of isoflavonoid synthesis (Sajid et al., 2022). In general, various factors affect metabolite accumulation, including inorganic elements (Nguyen et al., 2023), drought (Liu et al., 2023), and other environmental or climatic factors (Hano et al., 2022). Therefore, it is important to improve the cultivation and harvesting schedules of *S. japonica* (Yang et al., 2023).

## 5 Conclusion

In this study, variations in inorganic and flavonoid metabolites at the five developmental stages of *S. japonica* flowers were investigated. Multiple statistical analyses of the components were conducted to determine the relationship between FS quality and maturity. The accumulation of 16 inorganic elements and 173 flavonoid metabolites significantly changed during flower maturation. Enzymatic activity is a key factor affecting the conversion and accumulation of metabolites. The concentration of inorganic elements and harvesting schedule, as external factors interfering with enzyme activity, may alter the final quality of the FS. This study provides a novel perspective for revealing the quality formation patterns of *S. japonica* flowers. Understanding the relationship between flower maturity and quality helps in identifying the optimal harvesting period for *S. japonica*. Although inorganic elements have been speculated to affect flavonoid metabolism and synthesis in FS, the underlying mechanisms remain unclear; thus, further exploration of model plants should be conducted in future research.

## Data availability statement

The original contributions presented in the study are included in the article/**Supplementary Material**. Further inquiries can be directed to the corresponding author.

## Author contributions

JW: Funding acquisition, Project administration, Supervision, Writing – review & editing. TW: Formal Analysis, Methodology, Validation, Visualization, Writing – original draft. JT: Data curation, Methodology, Writing – original draft. LL: Conceptualization, Investigation, Resources, Writing – review & editing. YY: Data curation, Visualization, Writing – original draft. XZ: Investigation, Project administration, Supervision, Writing – review & editing.
