# Supplementary material for: Combined analysis of inorganic elements and flavonoid metabolites reveals the relationship between flower quality and maturity of Sophora japonica L
Source: Front Plant Sci. 2023 Nov 17;14:1255637. doi: 10.3389/fpls.2023.1255637 (PMC10691490; doi:10.3389/fpls.2023.1255637)
Supplement: Supplementary file 1 [file DataSheet_1.pdf]

Supplementary Material

Combined Analysis of Inorganic Elements and Flavonoid Metabolites Reveals the Relationship between Flower Quality and Maturity of *Sophora japonica* L.

Tian-Wang Wang[1,2], Jun Tan[1,2], Long-Yun Li[1,2], Yong Yang[1], Xiao-Mei Zhang[1*], Ji-Rui Wang[1,2*]

*** Correspondence:**
Ji-Rui Wang
wangjiruizyy@163.com

Xiao-Mei Zhang
ZXM761@163.com

1 Supplementary Figures

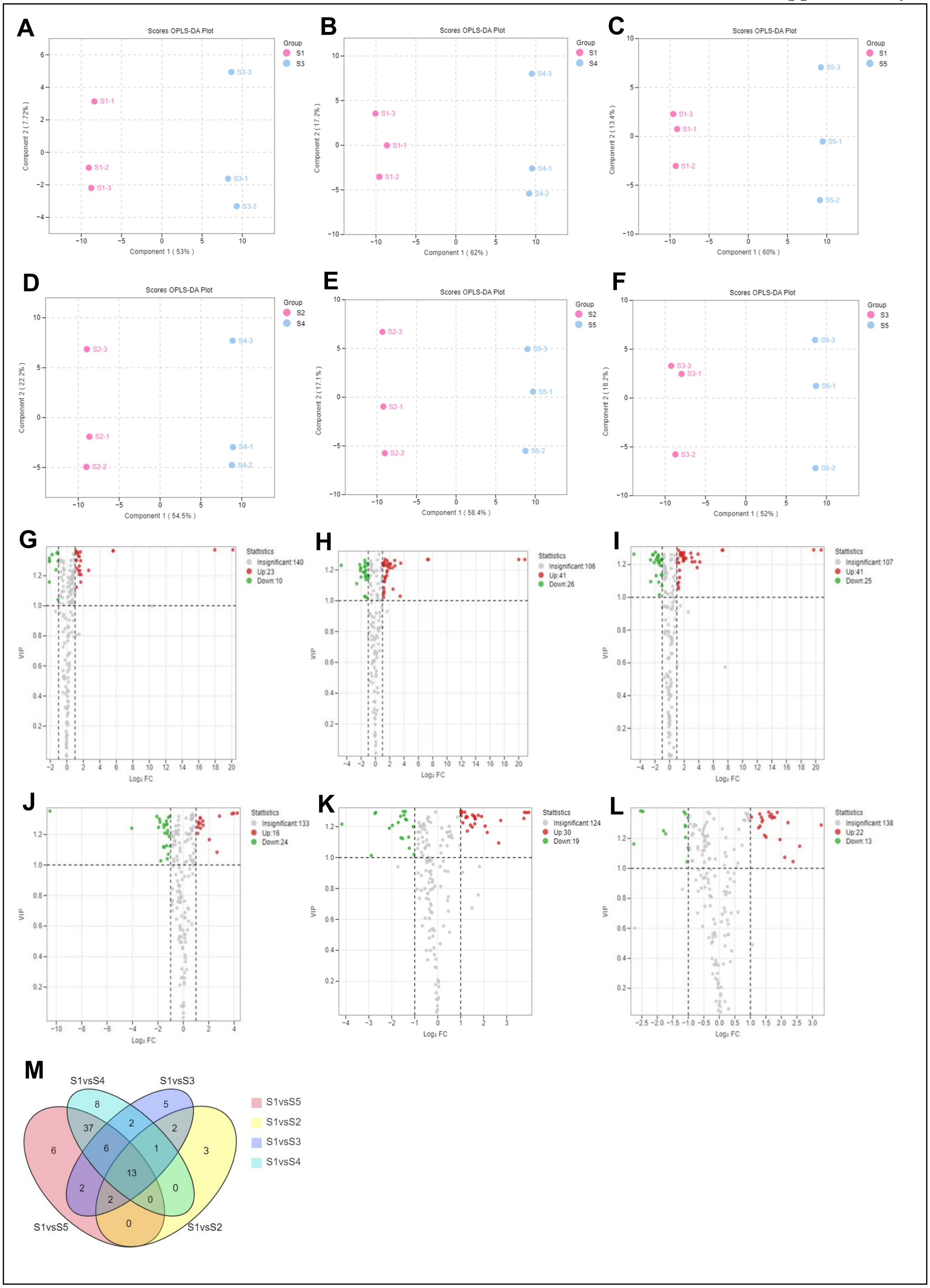

Supplementary Figure 1. Differential flavonoid metabolite analysis of the samples from two non-adjacent maturity periods of *S. japonica*. **(A−F)** Orthogonal partial least squares–discriminant

analysis (OPLS–DA) model plots for the comparisons among the five developmental stages (S1–S5) of FS: S1 vs. S3, S1 vs. S4, S1 vs. S5, S2 vs. S4, S2 vs. S5, and S3 vs. S5, respectively. **(G−L)** Volcano plots showing the expression levels of differential flavonoid metabolites in the comparisons S1 vs. S3, S1 vs. S4, S1 vs. S5, S2 vs. S4, S2 vs. S5, and S3 vs. S5, respectively. Red dots indicate upregulated, differentially expressed metabolites; green dots indicate downregulated, differentially expressed metabolites; and black dots indicate detected metabolites with insignificant differences in expression. **(M)** The Venn diagram shows the common and unique metabolites in S1 compared to the other groups.

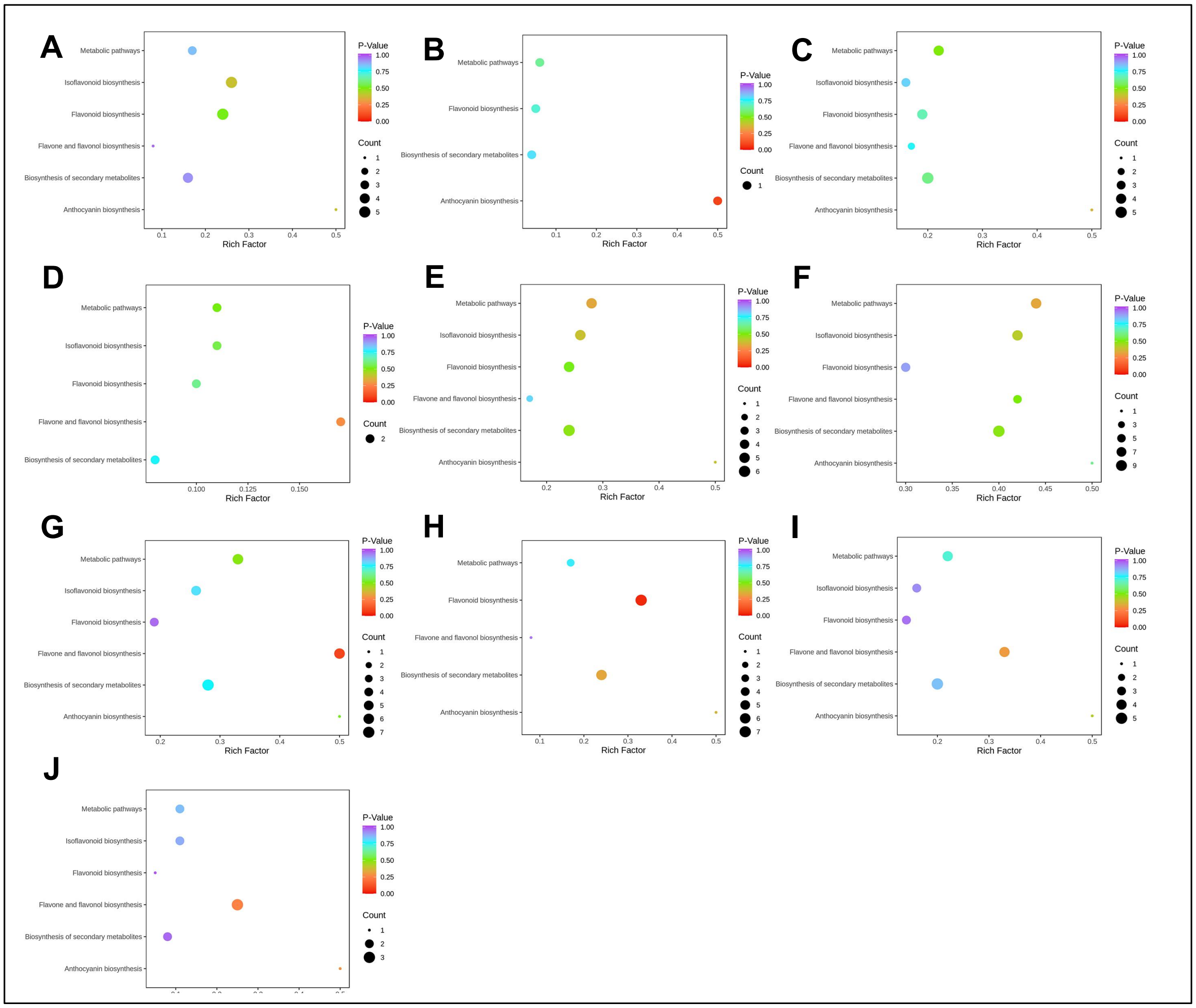

Supplementary Figure 2. Kyoto Encyclopedia of Genes and Genomes (KEGG) enrichment map of differential flavonoid metabolites KEGG enrichment map of differential flavonoid metabolites. **(A–D)** KEGG enrichment map of differential flavonoid metabolites from two adjacent flower maturity periods of FS: S1 vs. S2, S2 vs. S3, S3 vs. S4, and S4 vs. S5, respectively. **(E–J)** KEGG enrichment map of differential flavonoid metabolites from non-adjacent maturity periods of FS: S1 vs. S3, S1 vs. S4, S1 vs. S5, S2 vs. S4, S2 vs. S5, and S3 vs. S5, respectively.

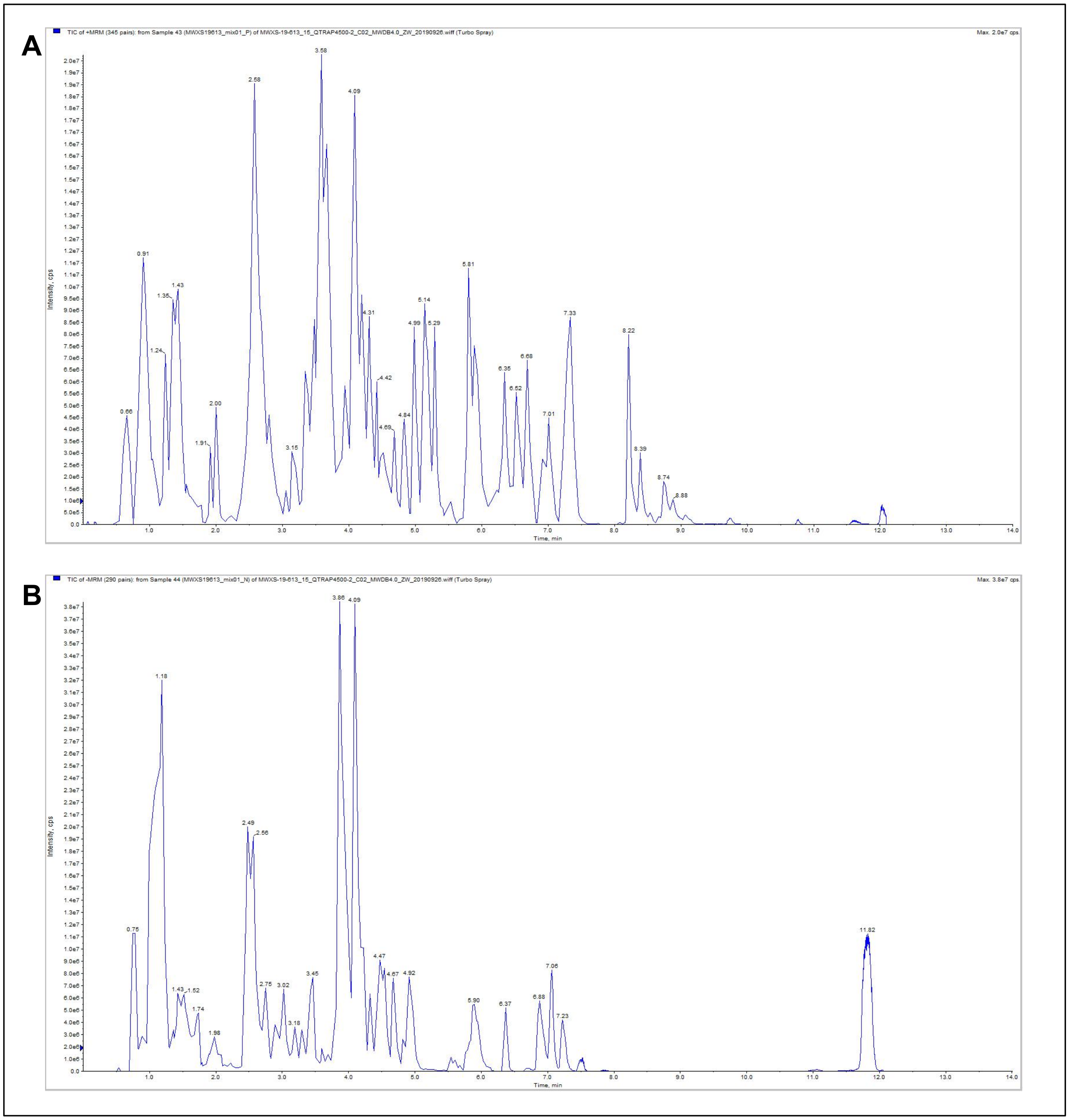
A
TIC of +MRM (345 pairs): from Sample 43 (MWXS19613_mix01_P) of MWXS-19-613_15_QTRAP4500-2_C02_MWDB4.0_ZW_20190926.wiff (Turbo Spray)
Max. 2.0e7 cps.
Intensity, cps
Time, min
B
TIC of -MRM (290 pairs): from Sample 44 (MWXS19613_mix01_N) of MWXS-19-613_15_QTRAP4500-2_C02_MWDB4.0_ZW_20190926.wiff (Turbo Spray)
Max. 3.8e7 cps.
Intensity, cps
Time, min

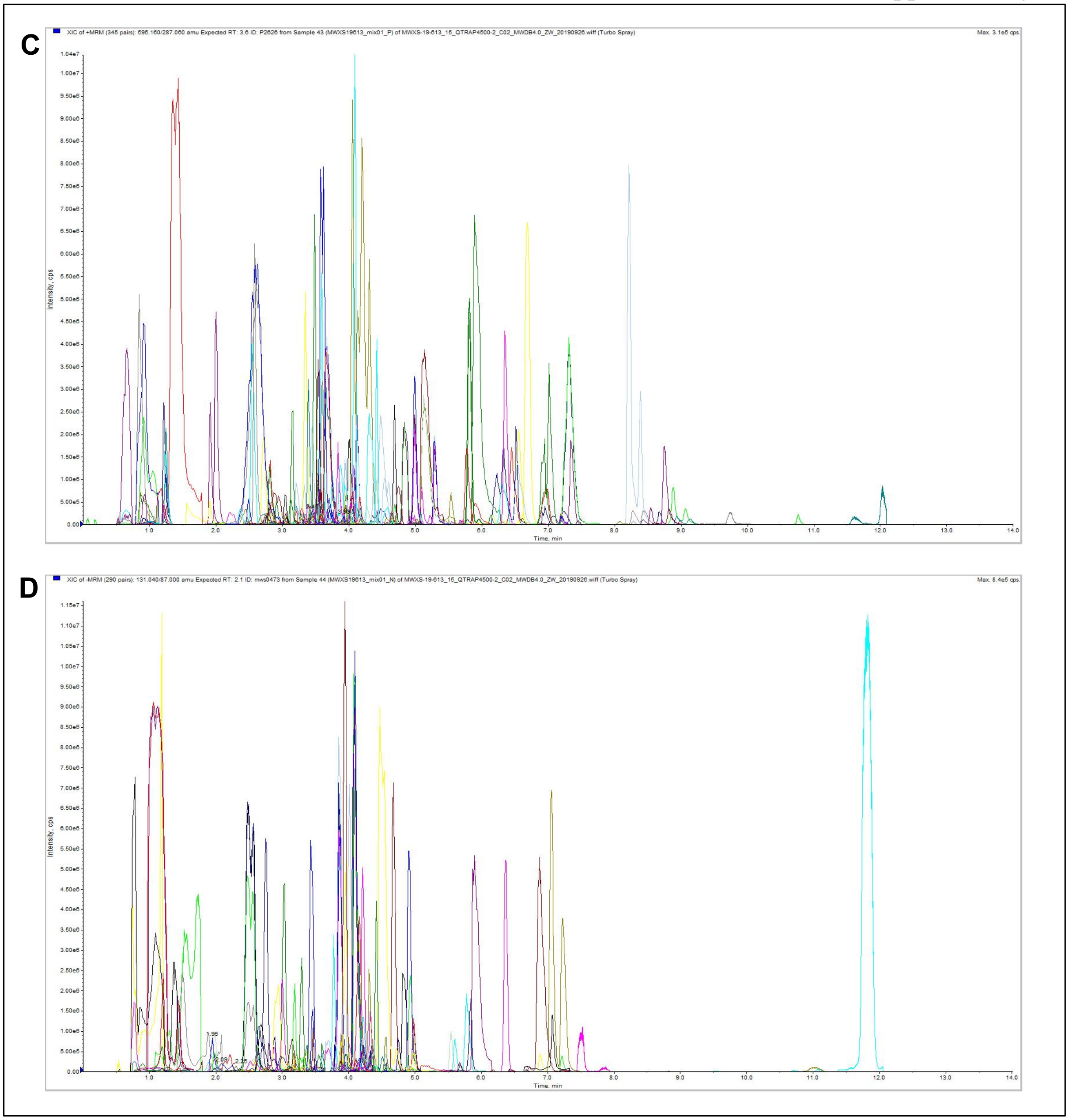

Supplementary Figure 3. Total ion current (TIC) diagram of the quality control samples in **(A)** electrospray ionization (ESI)$^+$ and **(B)** in ESI$^-$ mode. Multi-peak detection plot of the metabolites in multiple reaction monitoring (MRM) in **(C)** ESI$^+$ mode and **(D)** ESI$^-$ mode.

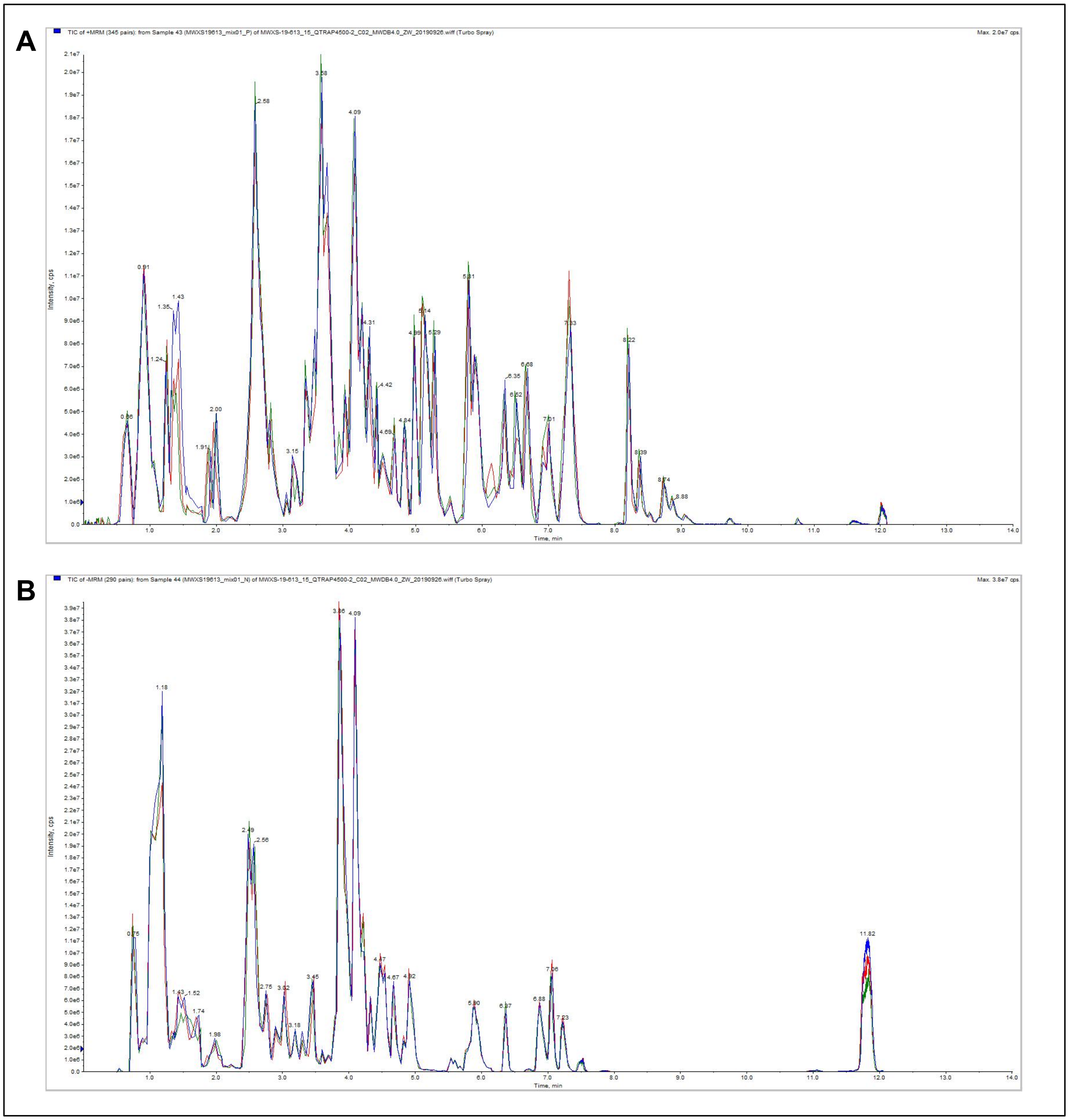

Supplementary Figure 4. Total ion current (TIC) overlaps of the three quality control samples using mass spectrometry detection. **(A)** TIC overlay plot in electrospray ionization (ESI)$^+$ mode. **(B)** TIC overlay plot in ESI$^-$ mode.

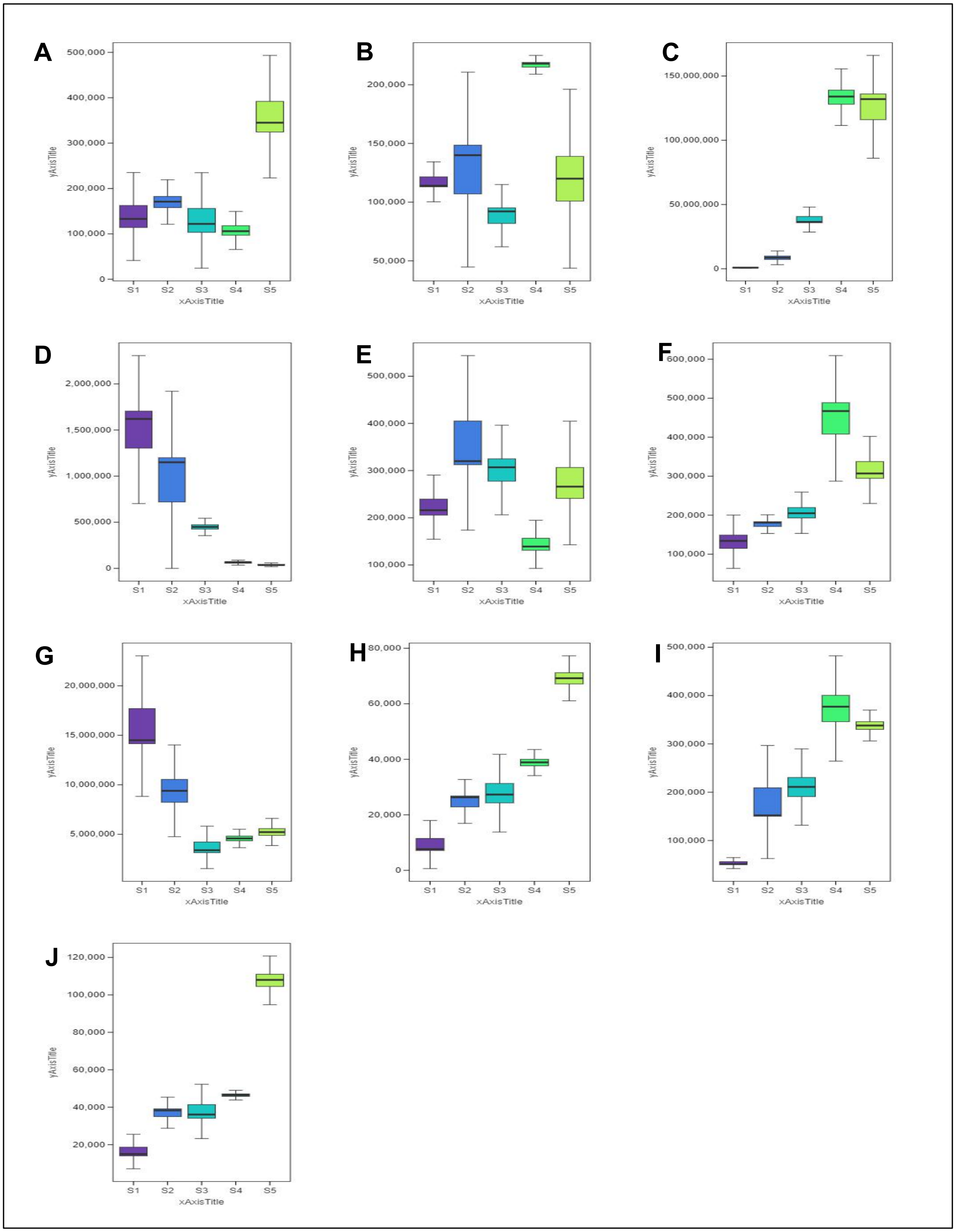

Supplementary Figure 5. Accumulation of ten flavonoid components over five periods **(A)** 3,7-Di-O-methyl quercetin, **(B)** luteolin, **(C)** cyanidin-3-O-glucoside, **(D)** eriodictyol, **(E)** genistein 7-O-beta-D-glucoside, **(F)** (-)-epigallocatechin, **(G)** sissotrin, **(H)** daidzein, **(I)** malonyldaidzin, **(J)** 7,4'-dihydroxyflavone.

2 Supplementary Tables

Supplementary Table 1. Microwave digestion procedure.

Supplementary Table 2. Inductively coupled plasma–optical emission spectrometry detection procedure.

Supplementary Table 3. A total of 173 flavonoid metabolites were characterized in the five maturity stages of *S. japonica*.

Supplementary Table 4. K-means cluster analysis of flavonoid metabolites in five maturity stages.

Supplementary Table 5. Differential flavonoid metabolites in adjacent developmental stages of *S. japonica*.

Supplementary Table 6. Differential flavonoid metabolites in non-adjacent developmental stages of *S. japonica*.

Supplementary Table 7. Dried weight of Flos Sophorae in the five developmental stages.

The detailed information can be found in a file called '*Table 1*'.
